# Visualization and Analysis in the Field of Pan-Cancer Studies and Its Application in Breast Cancer Treatment

**DOI:** 10.3389/fmed.2021.635035

**Published:** 2021-02-17

**Authors:** Xianwen Zhang, Han Lai, Fan Zhang, Yixi Wang, Li Zhang, Ni Yang, Chunrong Wang, Zheng Liang, Jieping Zeng, Jinrong Yang

**Affiliations:** ^1^School of Basic Medical Sciences, Chengdu University of Traditional Chinese Medicine, Chengdu, China; ^2^School of Foreign Languages, Chengdu University of Traditional Chinese Medicine, Chengdu, China; ^3^Department of General Surgery, The 5th Affiliated Hospital, Guangzhou Medical University, Guangzhou, China; ^4^Guangdong Provincial Key Laboratory of Stomatology, Guanghua School of Stomatology, Hospital of Stomatology, Sun Yat-sen University, Guangzhou, China; ^5^Department of Ophthalmology, Affiliated Hospital of Chengdu University of Traditional Chinese Medicine (Sichuan Provincial Hospital of Traditional Chinese Medicine), Chengdu, China

**Keywords:** pan-cancer, TCGA, bibliometrics, Scopus, VOSviewer

## Abstract

Although all cancers are molecularly distinct, many share common driver mutations. Pan-cancer analysis, utilizes next-generation sequencing (NGS), pan-cancer model systems, and pan-cancer projects such as The Cancer Genome Atlas (TCGA), to assess frequently mutated genes and other genomic abnormalities that are common among many cancer types, regardless of the tumor origin, providing new directions for tumor biology research. However, there is currently no study that has objectively analyzed the results of pan-cancer studies on cancer biology. For this study, 999 articles on pan-cancer published from 2006 to 2020 were obtained from the Scopus database, and bibliometric methods were used to analyze citations, international cooperation, co-authorship and keyword co-occurrence clusters. Furthermore, we also focused on and summarized the application of pan-cancer in breast cancer. Our result shows that the pan-cancer studies were first published in 2006 and entered a period of rapid development after 2013. So far, 86 countries have carried out international cooperation in sharing research. Researchers form the United States and Canada have published the most articles and have made the most extensive contribution to this field, respectively. Through author keyword analysis of the 999 articles, TCGA, biomarkers, NGS, immunotherapy, DNA methylation, prognosis, and several other keywords appear frequently, and these terms are hot spots in pan-cancer studies. There are four subtypes of breast cancer (luminalA, luminalB, HER2, and basal-like) according to pan-cancer analysis of breast cancer. Meanwhile, it was found that breast cancer has genetic similarity to pan-gynecological cancers, such as ovarian cancer, which indicates related etiology and possibly similar treatments. Collectively, with the emergence of new detection methods, new cancer databases, and the involvement of more researchers, pan-cancer analyses will play a greater role in cancer biology research.

## Introduction

The projected incidence of cancer was estimated to be more than 4,000,000 in China in 2020, with much larger numbers worldwide. Despite impressive progress made recently in cancer research (1, 2), there is an unmet need for effective treatments. Therefore, a more comprehensive understanding of the molecular characteristics and gene mutations of tumors is imperative (3). Generally, cancer is classified (4) according to the tissue where it occurs, such as in breast cancer, gastric cancer, or liver cancer. Most studies on the molecular, pathological, and clinical characteristics of tumors are based on the classification of tumor type. With reference to the guidelines followed by the oncology department of most cancer centers, principles of medicine or surgery for tumors are based on the origin (tissues and organ) (5). This framework has been established for a long time, but molecular analysis suggests that it could be problematic (6, 7): the tumors from different organs may have many common characteristics, whereas tumors from the same organ may have many differences. To investigate cancer study by molecular characteristics, medical researchers launched the Pan-Cancer Initiative (8) at a conference in Santa Cruz, California in 2012.

Pan-cancer analysis utilizes next-generation sequencing (NGS), pan-cancer model systems, and pan-cancer projects, such as The Cancer Genome Atlas (TCGA), to assess frequently mutated genes and other genomic abnormalities common that are common among many cancer types, regardless of the tumor origin (9). Currently, data for pan-cancer studies are obtained primarily from TCGA (10–12), which stores the sequences of all genes that encode proteins from more than 30 cancers that are represented by up to 500 tumors each. The results of pan-cancer analysis show that some cancers originating from different organs have molecular similarities, whereas some cancers originating from the same tissue may have very different genomic profiles (13, 14). Thus, researchers have begun classifying tumors into subtypes on the basis of pan-cancer data (15–17). For example, different subtypes of breast ductal carcinoma (luminalA, luminalB, HER2, and basal-like) can be identified by different biomarkers that manifest as different clinical characteristics (18), which should influence the treatment regimen patients should follow. Although increasing research on pan-cancer is gaining influence, there is no systematic analysis of its current hot spots, future trends, shortcomings, or impact on tumor biology.

With the aim of exploring the characteristics and advantages of pan-cancer research as well as promoting and guiding the involvement of researchers in pan-cancer research, this study evaluated the field of pan-cancer using visualization and bibliometrics analyzes. Bibliometric methods were used on 999 studies on pan-cancer published from 2006 to 2020, which were obtained from the Scopus database, to analyze the citations, international cooperation, co-authorship, and keyword co-occurrence clusters. Using breast cancer as an example, we explore how pan-cancer analysis can be used to identify new biomarkers and develop treatment options on the basis of the molecular subtypes. Collectively, this study provides interesting data to promote cancer biology research through exploring advancements in pan-cancer studies.

## Methods

### Source Database

This study used Scopus (https://www.scopus.com/), which is one of the most comprehensive databases for bibliometric analysis. Scopus is the world's largest database of abstracts and citations of peer-reviewed literature (scientific journals, books, and conference proceedings). The database includes different types of literature and relevant information that are critical to our study.

### Search Design

The title, abstract, and keywords related to pan-cancer of various types of studies were searched. Scopus was used as the only database. We designed the search query string as: (TITLE-ABS-KEY (“pan-cancer”) and limited the language to English. The search was conducted on October 20, 2020, so any updates to the database since then may reflect differences in the data reported herein. The search query returned 1,015 studies. Statistical analyzes of the country of publication, number of annual publications, type of publication, and t total citations were performed.

### Data Collection and Filtration

**Figure 1:**
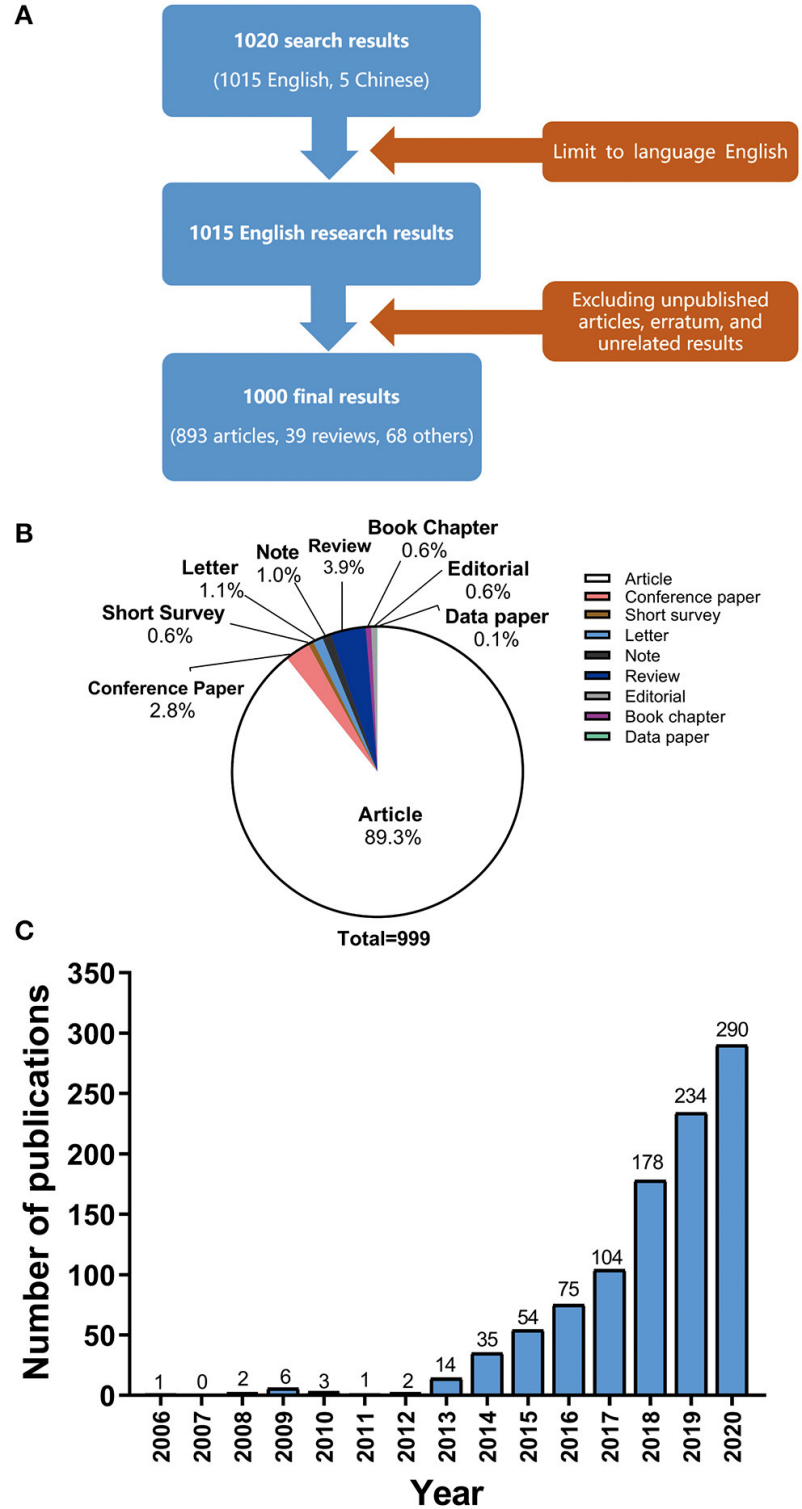
**(A)** Article types and proportion of all 999 publications related to pan-cancer studies. **(B)** Number of publications on pan-cancer studies from 2006 to 2020.

All queries using the above string and output were searched on Scopus with as much relevant information as possible included in CSV file. As shown in Figure 1A, unpublished articles, erratum, and unrelated results were excluded first. Then the two authors manually screened each title, abstract, and set of keywords to determine if any were significantly correlated with pan-cancer. For uncertain articles, we read the full text to determine whether to include them. Overall, 999 results (Figure 1A) were retained after independent screening, effective comparison, and discussion by two authors.

### Data Analysis

GraphPad Prism 8.0.2 software was used to conduct statistical analysis on the article type, number of articles and citations, and to draw pie charts and histograms from the results. VOSviewer, which is a scientific knowledge mapping software tool, uses “network data” (mainly literature knowledge units) for relationship building and visual analysis to perform scientific knowledge mapping and to show the structure, evolution, cooperation and other relationships (19). Co-authorship, co-citation, and keyword co-occurrence analyses were performed via VOSviewer1.6.13 after the CVS file containing the 999 publications was imported. In our study, various analytical results were presented in the form of network visualization maps through co-occurrence analysis of the national cooperation, author co-authorship, co-citation and author keywords.

### Research Ethics

All data in the study were collected from the Internet, and a bibliometric analysis was conducted. No animal or human subjects were involved in the study; thus, the approval of the ethics committee and the consent procedure were not required.

## Results

### Analysis of the Types of Study and Citations

Of these 999 different studies (Figure 1B), articles made up the largest portion, accounting for 89.3% (893 studies). Reviews accounted for 3.9% (39 studies), second only to articles. Other types of documents made up the remaining 6.8%, including the short surveys (0.6%, 6 studies), letters (1.1%, 11 studies), conference papers (2.8%, 28 studies), editorials (0.6%, 6 studies), notes (1%, 10 studies), book chapters (0.6%, 6 studies), and data paper (0.1%, 1 studies). It is worth noting that the publications of pan-cancer studies are mainly research articles. The data paper, which is a new type of document, is a peer-reviewed document that describes a dataset.

The annual number of published articles of pan-cancer studies is shown in Figure 1C. Since 2013, the number of publications on pan-cancer research has been increasing each year. Table 1 shows the top 10 journals with the highest number of pan-cancer publications. Some journals have high impact factors, such as Nature Communications (51 articles), Nucleic Acids Research (20 articles), and Nature Genetics (19 articles). A substantial number of articles were categorized as having high impact factors, which indicates that research on pan-cancer is a popular topic at present.

**Table 1:** The top 10 journals in the number of pan-cancer publications.

**Rank**	**Journal**	**NA**	**NC**	**CA**	**IF**	**Publisher**
1	Nature Communications	51	1,445	28.3	12.121	Nature Research
2	Scientific Reports	41	677	16.5	3.998	Nature Publishing Group
3	Oncotarget	30	471	15.7	0	Impact Journals LLC
4	Cancers	21	36	1.7	6.126	MDPI AG
5	Nucleic acids research	20	599	29.9	11.501	Oxford University Press
6	Nature Genetics	19	4,310	226.8	27.603	Nature Publishing Group
7	Bioinformatics	19	273	15.2	5.610	BioMed Central Ltd.
8	BMC Genomics	18	207	12.2	3.594	BioMed Central Ltd.
9	PLoS ONE	17	874	58.3	2.740	Public Library of Science
10	Cell Reports	15	109	7.8	8.109	Elsevier

*Rank, rank by NA; NA, number of articles; NC, number of citations; CA, Citations per article; IF, 2019 impact factor*.

**Figure 2:**
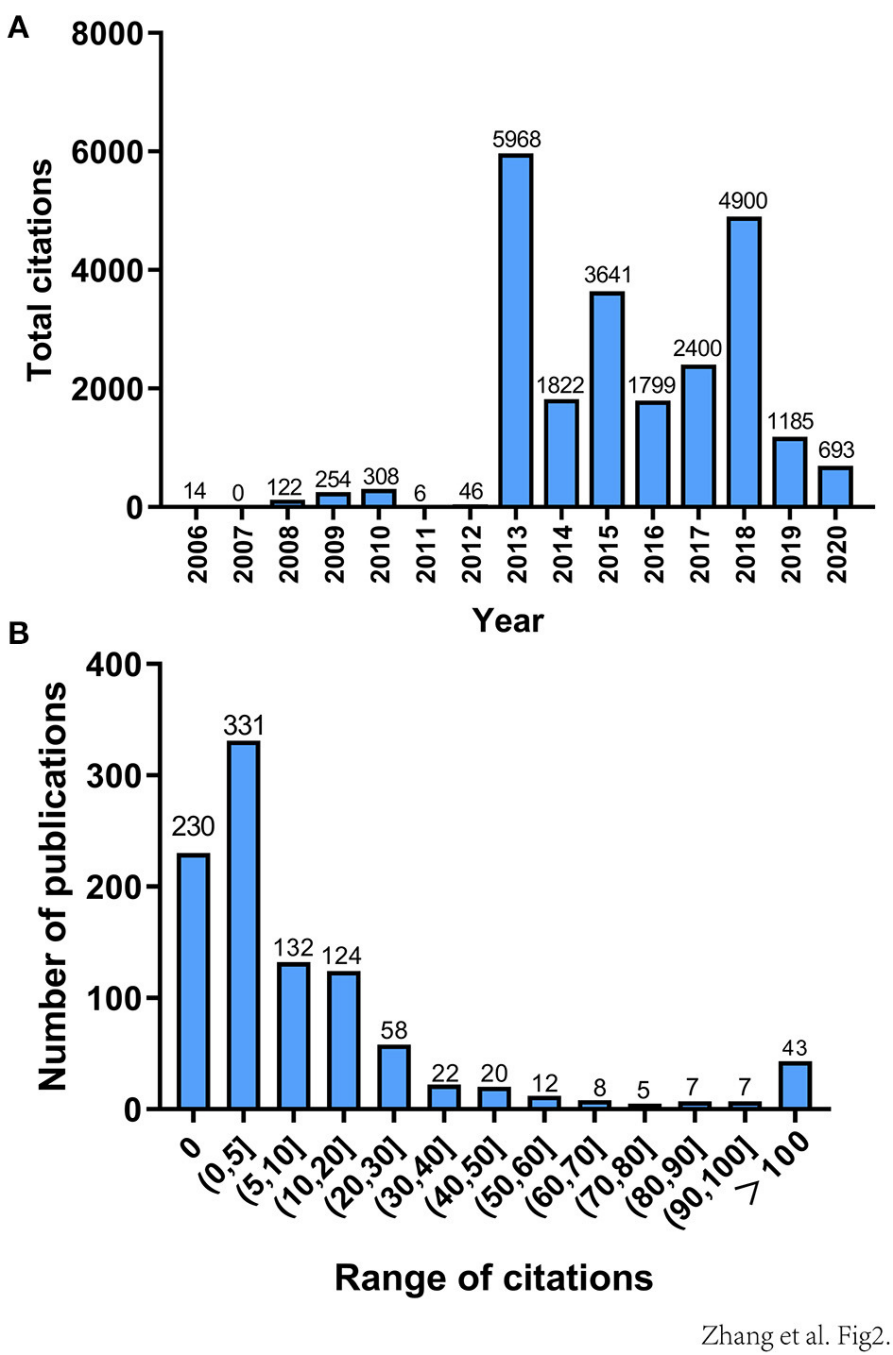
**(A)** Total annual citations of all 999 pan-cancer studies from 2006 to 2020. **(B)** Citation distributions of the 999 publications included. The range of citations (from 10 to 100) is divided into equally wide intervals, except those articles that received more than 100 citations and <10 citations.

However, the total number of citations decreased significantly each year compared to the number of publications in previous years (Figure 2A). We theorized that the total number of papers published increased, whereas in the total number of citations decreased, because older research has been accumulating citations since it was published, whereas newer research has had less time to accumulate citations. The range of citations from 10 to 100 could be divided into equal intervals, except for those articles that received more than 100 citations and <10 citations. The number of studies in each interval is counted (Figure 2B). There were 331 articles cited in the range of (0, 5), which is relatively the largest number; and despite the number of highly cited articles being small, research in this field is growing rapidly.

### Analysis of Country and International Cooperation

**Figure 3:**
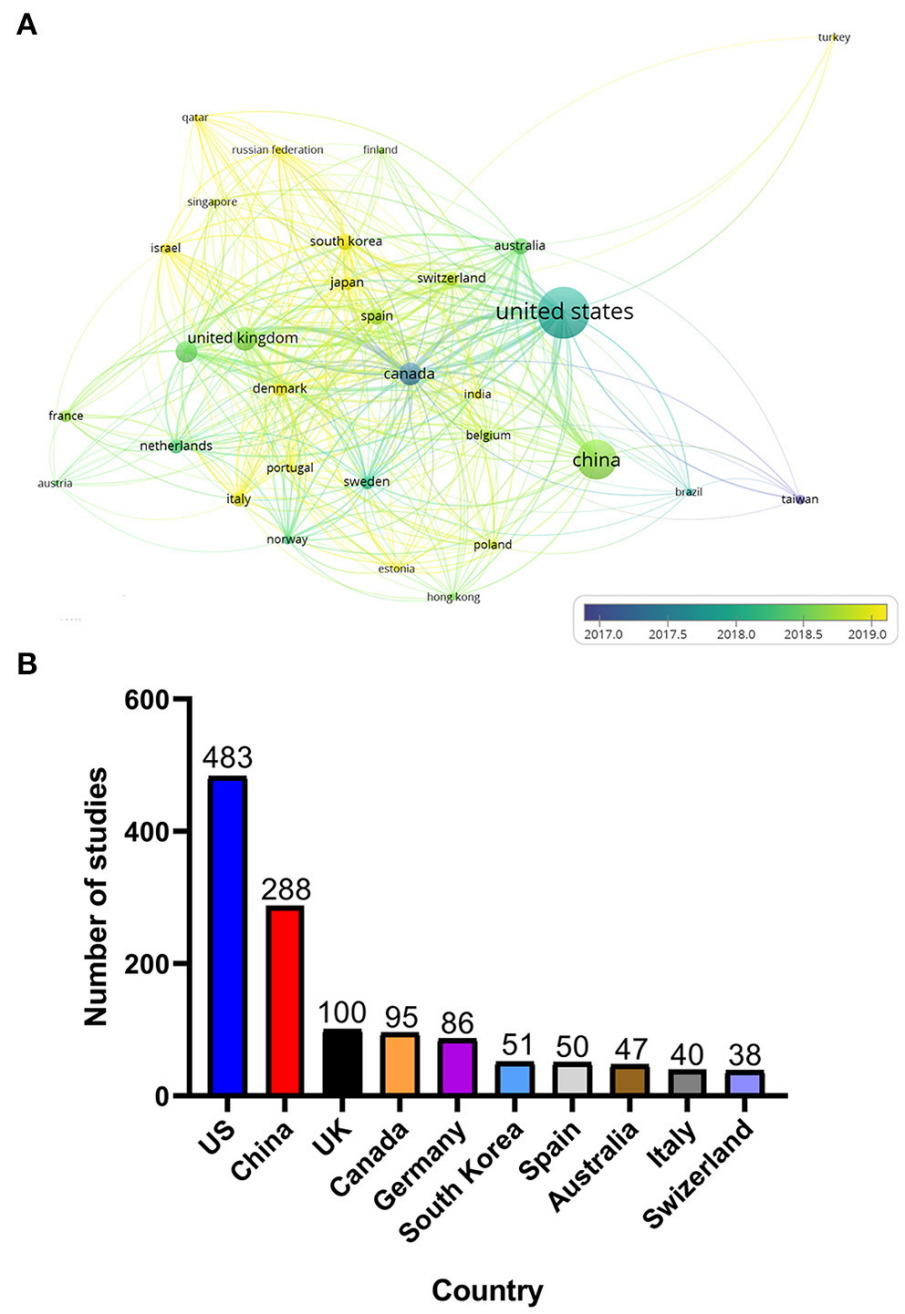
**(A)** Country co-authorship overlay visualization map. The size of each circle indicates the number of articles of that country. The distance between any two circles indicates the relatedness of their co-authorship link, and the thickness of the connecting line indicates the strength of the link. The color of each circle indicates the average years of publication of the articles from each country, according to the color gradient in the lower right corner. **(B)** Top 10 countries in terms of number of publications on pan-cancer studies.

The 999 studies included spanned 86 countries. However, when setting the minimum number of publications and citations per country to five, only 34 countries met the threshold. Figure 3A shows the different degrees of cooperation of 34 countries and the average publication date of each country's studies. Figure 3B shows the number of articles published by the top 10 countries for pan-cancer studies. Some research publications were carried out by researchers from two or more countries, so we counted the number of publications from those countries separately. The United States contributed more publications than did any other country, accounting for almost half of all pan-cancer publications. Canada was one of the first countries to begin pan-cancer research and has established collaboration with researchers from many other countries. In addition, researchers from China, Britain, Germany and South Korea have become increasingly active in recent years, which has revitalized the development of pan-cancer research.

### Co-Authorship Analysis and Author Co-Citation Analysis

**Figure 4:**
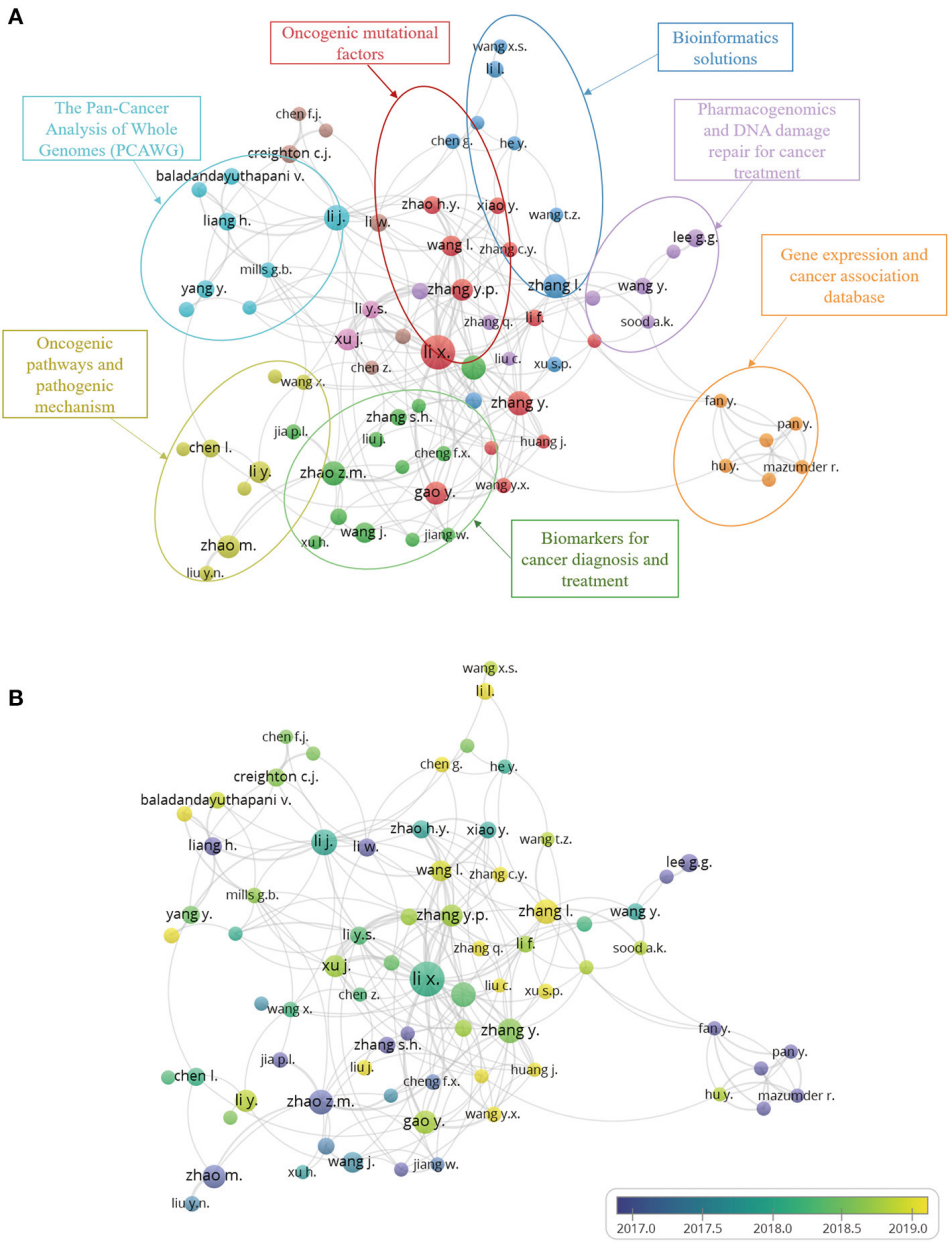
**(A)** Author co-authorship network visualization map. The same color represents a close relationship between the authors of clusters. The main research categories of each cluster were marked. **(B)** Author co-authorship overlay visualization map. The colors of different authors are based on the average year they published their articles.

According to VOSviewer analysis, a total of 5,558 authors were involved in the articles analyzed here, but only 95 authors met the minimum publication and citation thresholds when set to four. During the analysis, we found many similar or identical abbreviations of names, for which we manually supplemented most of the names by referring to the original text. The number of publications and collaborations among authors can be seen from the overlay visualization map of the author co-authorship analysis (Figure 4A). The same color represents a closely related cluster of authors; the size of the circle represents the number of publications; and the thickness of the line represents the closeness of cooperation between the authors. Meanwhile, by analyzing the articles in each cluster, we manually and subjectively identified and labeled the main areas of research for each cluster. The orientations of pan-cancer studies include the following: (1) mechanism of tumor by oncogenic mutational factors; (2) oncogenic pathways and pathogenic mechanism; (3) different cancer biomarkers for cancer diagnosis and treatment; (4) pan-cancer analysis of whole genomes (PCAWG) and bioinformatics solutions; (5) Pharmacogenomics and DNA damage repair for cancer treatment; and (6) gene expression and cancer association database. Figure 4B depicts the co-authorship overlay visualization map, in which the colors of different authors are based on the average year of their publications. Mazumder R, Zhao ZM, Lee GG, et al. are represented by purple, which indicates indicating that they began their research in this field at an earlier date. Newer researchers, like Zhang L., Huang J., and others, are shown in yellow.

**Figure 5:**
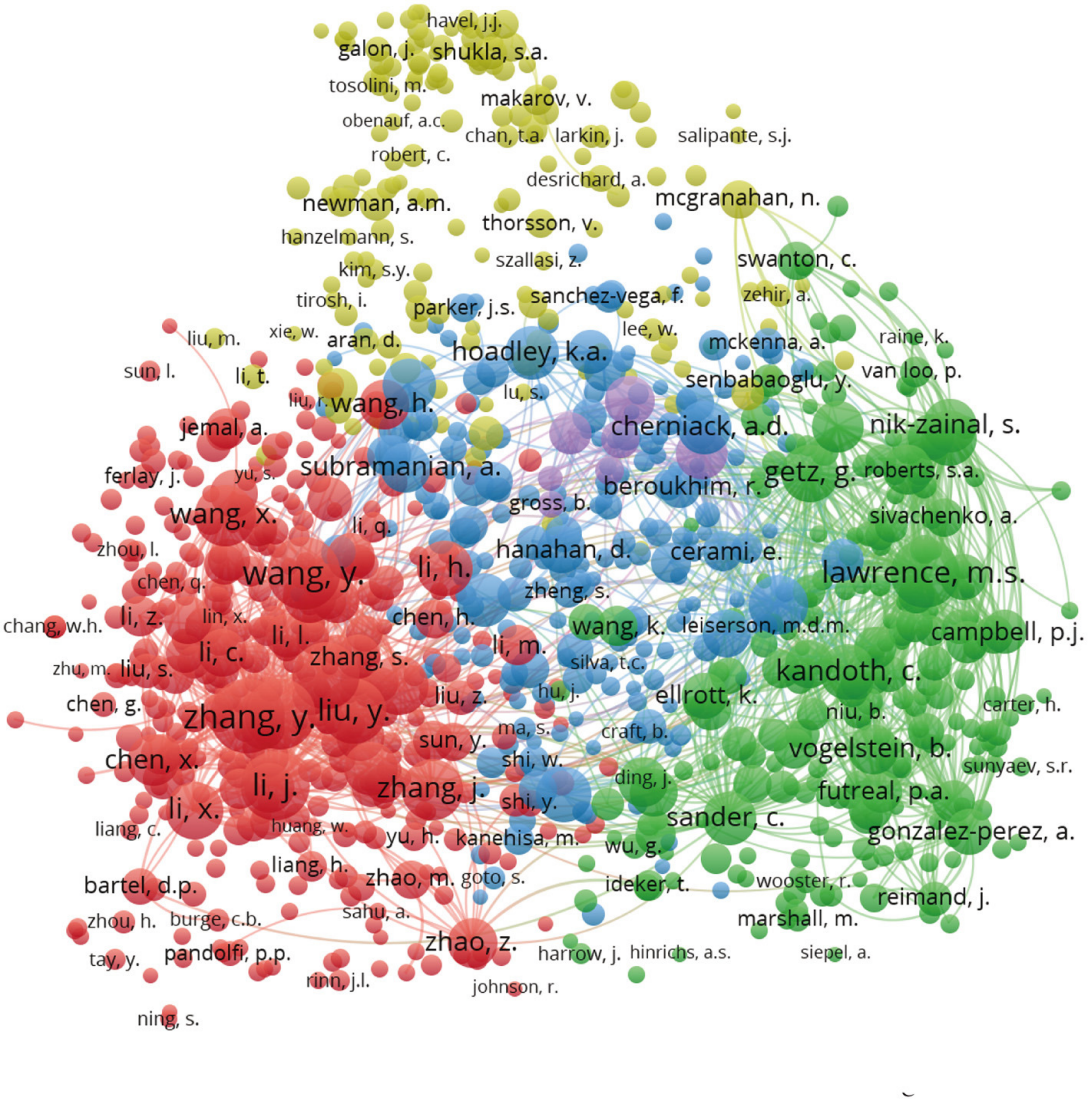
Author co-citation network visualization map. The area of circles indicated the total citations of each author. Different colors of the circles represented clusters divided by co-citations. Authors in the same color are based on the close relationship connected groups.

For author co-citation analysis (20, 21), when the articles of two authors were cited by a third author in the same publication, the two authors were said to have a co-citation relationship. The higher the co-citation frequency between two authors, the closer their academic relationship. In the author co-citation analysis, the threshold was set at a minimum of 20 citations, and 988 of the 5,558 authors met the threshold. The size of each circle represents the number of times the author has been cited, so a larger circle indicates a higher number of citations. As shown in Figure 5, the author co-citation analysis network visualization map is divided into five colors (red, green, yellow, purple, and blue). Authors that have published on the same topic are represented by the same color, and authors with the same color are based on the connected groups. The figure visually demonstrates the impact of authors who have conducted pan-cancer research, thus, this map reveals the leading researchers in this field. Table 2 shows the top 10 articles that have been cited in pan-cancer studies. Most of the highly cited articles in the table are research articles with high impact factors.

**Table 2:** The top 10 articles cited in pan-cancer studies.

**Rank**	**Journal**	**Article title**	**Authors**	**CA**	**IF**	**Article type**	**Publication year**
1	Nature Genetics	The cancer genome atlas pan-cancer analysis project	Weinstein JN, Collisson EA, Mills GB, et al.	2,359	27.603	Research article	2013
2	Nature	Mutational landscape and significance across 12 major cancer types	Kandoth C, McLellan MD, Vandin F, et al.	2,087	42.778	Research article	2013
3	Nature Genetics	Pan-cancer patterns of somatic copy number alteration	Zack TI, Schumacher SE, Carter SL, et al.	855	27.603	Research article	2013
4	Nature Medicine	The prognostic landscape of genes and infiltrating immune cells across human cancers	Gentles AJ, Newman AM, Liu CL, et al.	790	36.130	Research article	2015
5	Cell	Oncogenic signaling pathways in the cancer genome atlas	Sanchez-Vega F, Mina M, Armenia J, et al.	402	38.637	Research article	2018
6	Nature Genetics	Pan-cancer network analysis identifies combinations of rare somatic mutations across pathways and protein complexes	Leiserson MDM, Vandin F, Wu H-T, et al.	369	27.603	Research article	2015
7	Cell Reports	Pan-cancer immunogenomic analyses reveal genotype-immunophenotype relationships and predictors of response to checkpoint blockade	Charoentong P, Finotello F, Angelova M, et al.	344	8.109	Research article	2017
8	Wspolczesna Onkologia	The Cancer Genome Atlas (TCGA): an immeasurable source of knowledge	Tomczak K, Czerwińska P, Wiznerowicz M.	322	0.215	Review	2015
9	Cell	An integrated TCGA pan-cancer clinical data resource to drive high-quality survival outcome analytics	Liu Jianfang, Lichtenberg T, Hoadley KA, et al.	313	38.637	Research article	2018
10	Cell	Cell-of-origin patterns dominate the molecular classification of 10,000 tumors from 33 types of cancer	Hoadley KA, Yau C, Hinoue T, et al.	304	38.637	Research article	2018

*Rank, rank by CA; CA, Citations per article; IF, 2019 impact factor*.

### Analysis of Keyword Co-Occurrence Clusters

**Figure 6:**
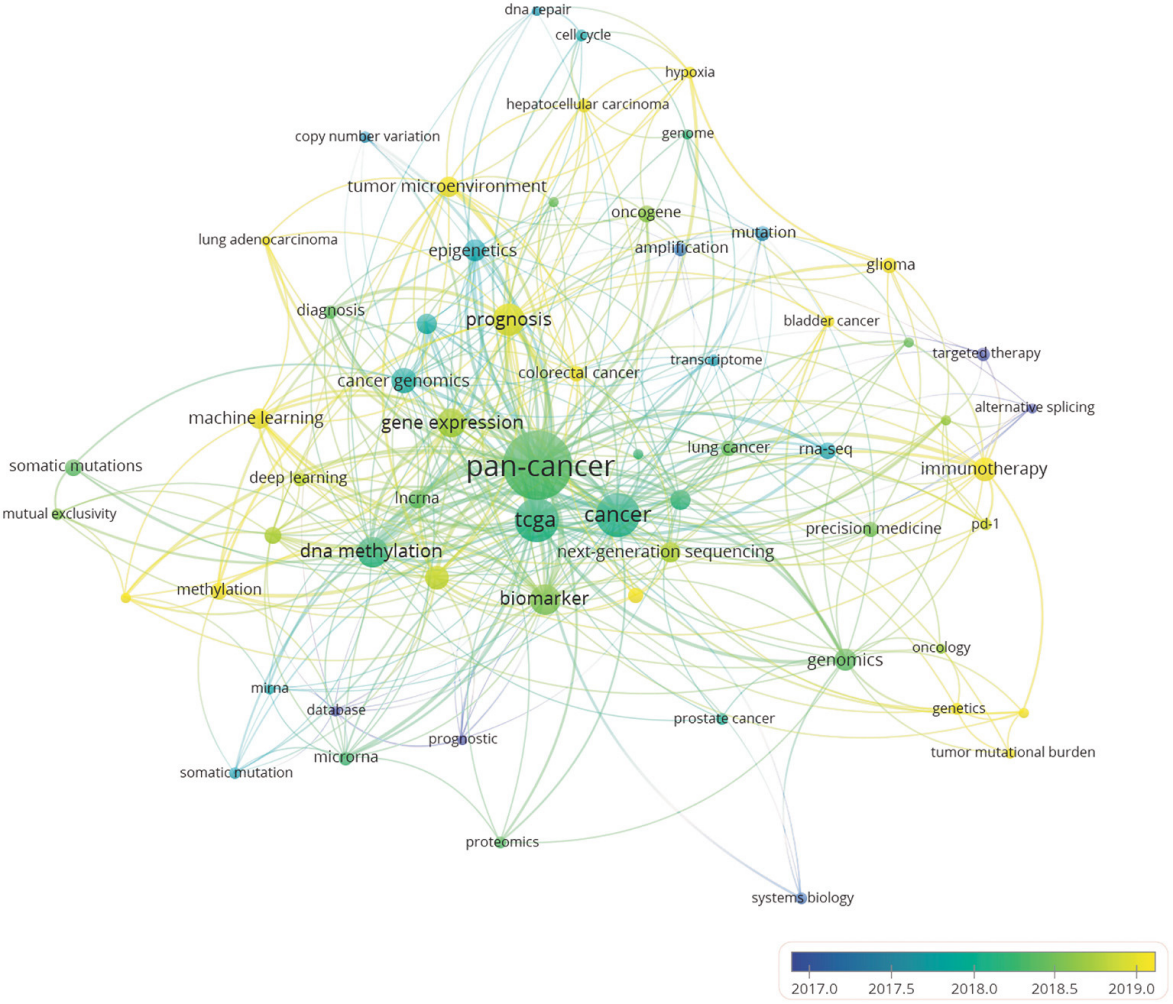
Author keyword overlay visualization map. The size of each circle indicates the frequency of occurrences of the author keyword. According to the color label in the lower right corner, the color of each circle indicates the average year when the keyword appeared in articles. The distance between any two circles is indicative of their co-occurrence link, and the thickness of the connecting line indicates the strength of the link.

An analysis of the keyword co-occurrence clusters (20) reveals the internal composition and structure of the academic field through a co-occurrence relationship based on keywords. The results can also be used to reveal the development trends of specialized disciplines. When the minimum number of occurrences of a keyword was set to 566 of the total 1,787 keywords met the threshold. The overlay visualization drawn in proportion to events (Figure 6) shows what content of pan-cancer studies is most popular. Meanwhile, author keywords are marked in different colors according to the average year of their publishing activities. Bibliometric analysis of the keywords can provide a simple description of the research hot spots. Owing to the non-unity of single and plural keywords and the synonyms in some publications, we altered some of the author keywords in CSV file, such as unifying “pan cancer” and “pan-cancer analysis” as “pan-cancer,” and unifying “the cancer genome atlas” as “TCGA,” so that these terms would be represented accurately in the data analysis. Keywords such as “targeted therapy,” “cancer genomics” and “epigenetics” appeared more frequently in the early stage. However, in recent years, terms such as “prognosis,” “immunotherapy,” “methylation,” “glioma” were more frequently used, which indicates that the research focus in the field has shifted from observing the molecular characteristics of major tumors to identifying molecular signatures for patient treatment and prognosis. Keywords, such as TCGA, biomarker, NGS, immunotherapy, DNA methylation, and prognosis, appear frequently in all author keywords, so these terms are presumed to be the hot spots of pan-cancer research.

## Discussion

Cancer has been recognized as a genetic disease, and the goal of the Pan-Cancer Initiative is to identify and analyze the genetic changes that contribute to the commonality and differences among the genotypes and phenotypes that determine tumor lineage (8, 21). By conducting extensive pan-cancer research, researchers can explore the potential genomes of cancer mutations, including germline mutation (genetics) and somatic mutation (acquisition), to identify biomarkers that aid in cancer diagnosis (22). Furthermore, as common molecular signatures and action signal pathways, are discovered, in the etiology and optimal therapeutics of that work for one tumor can be applied to another, tumor on the basis of its molecular subtype, which will help lead to various innovative cancer treatment methods. Tumors with low incidence, such as pediatric malignancies, benefit substantially (23).

Since the Pan-Cancer Initiative was launched in 2012, pan-cancer studies have entered a period of rapid development (Figure 1C). A growing number of researchers from various countries have initiated pan-cancer research. The fact that 2,400 authors have published more than one publication and that the predominance of articles are recent research (Figure 1B) indicates that pan-cancer research is gaining popularity among researchers. It is estimated that there will be more than 300 articles published on pan-cancer studies in 2020. Molecular analysis techniques of tumors, such as NGS, RNA-seq and DNA methylation arrays, have gradually matured and become affordable (24–26), which has supported further development of pan-cancer research. As shown in Figure 3, the United States, Canada and other developed countries with advanced technology and closer cooperation have carried out research over longer time periods, so they are leaders in this field. The number of pan-cancer research publications in China ranks second worldwide. Because of China's large population, high number of cancer cases, and high mortality rate, the Chinese have invested in research that could lead to the detection, diagnosis, and treatment of cancers. Meanwhile, the rapid growth of China's GDP has also provided support for China's pan-cancer studies. According to the keyword co-occurrence classification and timeline in Figure 4, we found that the previous study mainly used the TCGA database to distinguish similarities and differences between tumors of different types of tissue, whereas the current research direction is to explore mutated tumor biomarkers through tumor gene and cell molecular detection comparisons. The molecular characterization of many different tumors may help to identify biomarkers and corresponding immunotherapies that can be used to treat different cancers of the same molecular characterization in patients with other types of cancer. In the future, when the pan-cancer analysis data of most tumor have expanded, we predict that the focus of research on cancer will shift to specific molecular subtypes and previously unstudied tissues with the aim of developing tumor treatments, prognosis, prevention, and detection markers. By using NGS and TCGA, pan-cancer studies have made significant contributions to our understanding of DNA and RNA variants across many cancer types (27). However, most current pan-cancer studies are based on the 33 tumors that are registered in the TCGA database. Therefore, it is necessary to include new tumor types, integrate data from similar databases, and attract more researchers to conduct pan-cancer studies.

There are numerous pan-cancer studies on various tumors, especially breast tumors. Breast cancer is currently one of the most common cancers (28). The classification of breast cancer subtypes is based on molecular characteristics, genomic characteristics, clinical data, and histomorphology (29). A study published in Nature (18) analyzed 510 tumors from 507 patients by using genomic DNA copy number array, DNA methylation, exome sequencing, mRNAseq, microRNAseq, and antiphasing protein array and revealed four subtypes of breast cancer (luminalA, luminalB, HER2, and basal-like). At present, there are over-treatment and under-treatment for different subtypes of breast cancer, and primary routine surgery is no longer the best choice for all patients (30–32). On the basis of a standardized uPA/PAI-1 biomarker ELISA, almost half of node-negative breast cancer patients (negative) could be spared chemotherapy (33). According to different clinical tumor subtypes, treatment methods include endocrine therapy, anti-HER2 targeting and chemotherapy (34). Through a comprehensive analysis of all 33 TCGA tumor types, it was found that invasive breast cancer (BRCA) and highly malignant serous ovarian cystadenocarcinoma (OV), endometrial carcinoma (UCEC), cervical squamous cell carcinoma, cervical adenocarcinoma (CESC), and uterine carcinosarcoma (UCS) are similar at the molecular level (35). Except for OV, pan-gynecologic tumor types have similar miRNA profiles (36), which indicates that pan-gynecologic tumors have relative specificity (37). This also suggests that the diagnosis and treatment strategies for BRCA may be applied to other gynecologic cancers with high cure rates. Furthermore, the development of pan-cancer studies on breast cancer will also may lead to the diagnosis and treatment of rare gynecological cancers.

Collectively, through bibliometric analysis and visualization of 999 publications, our study analyzed the research hot spots, advantages, and disadvantages in the field of pan-cancer research, with the aim of providing references for future researchers who intend to conduct pan-cancer studies. Our results reveal that pan-cancer analysis and gene sequencing of the biomarkers and genes of different cancer types, especially in breast cancer research, can lead to molecular characterizations and subsequent identification of similar etiologies for which the therapeutics of one tumor type can be applied to other tumor types of a similar genomic profile. Thus, with the emergence of new sequencing methods and cancer databases, pan-cancer analysis will play an increasingly significant role in cancer biology research.

## Data Availability Statement

The original contributions presented in the study are included in the article/Supplementary Material, further inquiries can be directed to the corresponding authors.

## Author Contributions

XZ, HL, and YW did bibliometric analysis and wrote the manuscript. LZ, NY, and CW extracted the data and used VOSviewer to draw the map. FZ, ZL, JZ, and JY designed the research, organized the calculations, and contributed to manuscript writing and rivision. All authors have read and approved the final manuscript.

## Conflict of Interest

The authors declare that the research was conducted in the absence of any commercial or financial relationships that could be construed as a potential conflict of interest.
